# Live-cell fluorescence imaging to investigate the dynamics of plant cell death during infection by the rice blast fungus *Magnaporthe oryzae*

**DOI:** 10.1186/s12870-016-0756-x

**Published:** 2016-03-22

**Authors:** Kiersun Jones, Dong Won Kim, Jean S. Park, Chang Hyun Khang

**Affiliations:** Department of Plant Biology, University of Georgia, Athens, 30602 USA

**Keywords:** Biotrophic interfacial complex, Confocal microscopy, Fluorescein diacetate, Hemibiotrophy, Host-pathogen interactions, *Oryza sativa*, Plasmodesmata, Programmed cell death, Propidium iodide, Vacuole

## Abstract

**Background:**

Plant cell death plays important roles during plant-pathogen interactions. To study pathogen-induced cell death, there is a need for cytological tools that allow determining not only host cell viability, but also cellular events leading to cell death with visualization of pathogen development. Here we describe a live cell imaging method to provide insights into the dynamics of cell death in rice (*Oryza sativa*). This method uses live-cell confocal microscopy of rice sheath cells mechanically damaged or invaded by fluorescently-tagged *Magnaporthe oryzae* together with fluorescent dyes fluorescein diacetate (FDA) and propidium iodide (PI). FDA stains the cytoplasm of live cells exclusively, thus also visualizing the vacuole, whereas PI stains nuclei of dead cells.

**Results:**

We first demonstrated that confocal microscopy of rice leaf sheaths stained with FDA and PI discriminated between live cells and mechanically-killed cells. FDA-derived fluorescein was confined to the cytoplasm of live cells, indicating the intact vacuolar and plasma membranes. We also observed previously unreported fluorescein patterns in mechanically damaged cells. These patterns include: (1) homogeneous distribution of fluorescein in the increased area of the cytoplasm due to the shrunken vacuole; (2) the increase of the fluorescein intensity; and (3) containment of the brighter fluorescein signal only in affected cells likely due to closure of plasmodesmata. We refer to these as novel fluorescein patterns in this study. Simultaneous imaging of fluorescently-tagged *M. oryzae* (red) and FDA staining (green) in rice cells revealed characteristic features of the hemibiotrophic interaction. That is, newly invaded cells are alive but subsequently become dead when the fungus spreads into neighbor cells, and biotrophic interfacial complexes are associated with the host cytoplasm. This also revealed novel fluorescein patterns in invaded cells. Time-lapse imaging suggested that the FDA staining pattern in the infected host cell progressed from typical cytoplasmic localization (live cell with the intact vacuole), to novel patterns (dying cell with closed plasmodesmata with the shrunken or ruptured vacuole), to lack of fluorescence (dead cell).

**Conclusion:**

We have developed a method to visualize cellular events leading to host cell death during rice blast disease. This method can be used to compare and contrast host cell death associated with disease resistance and susceptibility in rice-*M. oryzae* and other host-pathogen interactions.

## Background

Plants are challenged by various pathogens, and plant cell death can be associated with disease resistance and susceptibility [1–4]. Hypersensitive cell death is a well-described resistance-associated cell death that occurs rapidly at the infection site and restricts the growth of certain pathogens [2, 4–8]. This cell death involves regulated processes leading to characteristic morphology of cell death, known as programmed cell death (PCD), which differs from accidental destruction of cellular integrity [2, 4, 8–10].

To study cell death in plant-pathogen interactions, several cytological methods have been commonly used in assessing host cell viability during infection. A dye exclusion method uses certain dyes, such as tryphan blue and SYTOX, that are excluded from the plasma membrane of live cells but stain internal components of dead cells [1, 11]. Another method is a sucrose-induced plasmolysis, in which live cells exhibit plasma membrane pulled away from the cell wall in the presence of a hypertonic solution such as 0.5 M sucrose [12, 13]. These methods are useful to determine whether host cell viability is correlated with promoting or restricting the growth of pathogens and also to define the lifestyle of pathogens such as biotrophy (acquiring nutrients from live host cells), necrotrophy (acquiring nutrients from dead host cells), or hemibiotrophy (acquiring nutrients from initially live host cells but later from dead host cells) [14]. However, these methods do not provide cytological details on the processes that precede cell death. Increasing evidence suggests that processes leading to host cell death contribute to disease resistance or susceptibility depending on the lifestyle of pathogens and that the vacuole is a key organelle in these processes [4, 15–18]. Therefore, to study pathogen-associated plant PCD, there is a need for a cytological tool that allows not only the determination of the host cell viability but also the characterization of cellular dynamics leading to cell death in the context of pathogen development.

**Fig. 1:**
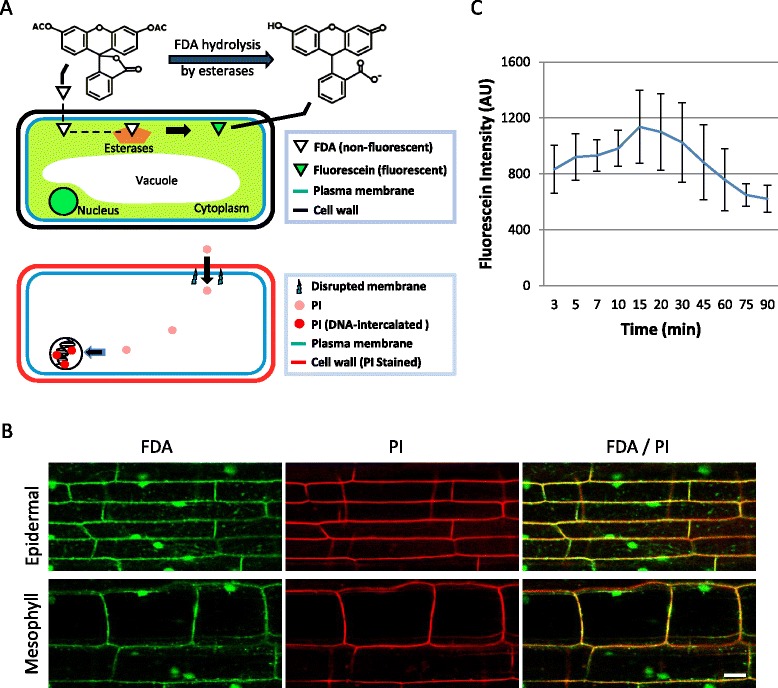
FDA and PI staining of plant cells. **a** Diagrams showing fluorescein diacetate (FDA) and propidium iodide (PI) staining of plant cells. Top: Non-fluorescent FDA molecules pass through the intact plasma membrane and are hydrolyzed by intracellular esterases to produce fluorescein. The membrane-impermeable fluorescein accumulates in the cytoplasm and exhibits green fluorescence. Bottom: In a non-viable cell with a disrupted plasma membrane, PI enters the cell and intercalates with DNA to form a bright red fluorescent complex in a nucleus. PI also stains the cell wall in both live and dead cells. **b** Single plane confocal images of rice sheath epidermal cells (top) and immediately underlying mesophyll cells (bottom) stained with both FDA (green) and PI (red). Bar = 20 μm. **c** Time-course average pixel intensity of FDA-stained rice sheath epidermal cells. Blue line is an average ± SD of intensity measurements of defined regions of cytoplasmic fluorescence (*n* = 6 at each time point). Fluorescein intensity peaked on average at 15 min after staining and then steadily declined

Visualization of a cell with fluorescent compounds is a useful tool for the analysis of cellular architecture and viability. FDA is a fluorogenic ester compound that passes through the plasma membrane and is hydrolyzed by intracellular esterases to produce a negatively charged membrane-impermeable fluorescein with green fluorescence [19–21] (Fig. 1a). FDA, therefore, can serve as a positive test assay for viable cells that are metabolically active [21]. In addition, FDA staining can also visualize vacuoles because negatively charged fluorescein selectively accumulates in the cytoplasm but is excluded from the vacuole [22]. Several studies have utilized FDA to visualize vacuoles such as in epidermal cells of *Pisum sativum* [23], trichomes of *Arabidopsis thaliana* [24] and guard cells of *Vicia faba* [25], but there is no report of FDA-based visualization of the vacuole dynamics in response to pathogens. While FDA stains the cytoplasm and visualizes vacuoles of viable cells, PI stains the nuclei of dead cells [26]. PI passes through damaged cell membranes and intercalates with DNA to exhibit bright red fluorescence (Fig. 1a). Since the dye is excluded by intact cell membranes, PI is an effective stain to identify dead cells. In addition, PI stains plant cell walls regardless of cell viability.

Rice blast is an economically important disease of rice caused by the blast fungus *Magnaporthe oryzae*. On the rice leaf surface, the fungus produces a specialized penetration cell called the appressorium to mechanically breach into an epidermal rice cell [27]. Cytological studies have documented hemibiotrophic fungal invasion and rice cell responses based on live-cell imaging of optically clear leaf sheaths of susceptible rice [12, 13, 18]. The fungus produces invasive hyphae (IH) that fill the first-invaded cells and then spread into neighbor cells. The initial invasion of the first cell and successive invasion of neighbor cells are biotrophic because invaded cells retain the ability to plasmolyze in response to a hypertonic solution. During the biotrophic invasion, IH are associated with biotrophic interfacial complexes (BICs) that are hypothesized to deliver effector proteins into the host cytoplasm across the extra-invasive hyphal membrane (EIHM) [28]. The effector proteins that reach the invaded cell’s cytoplasm move into adjoining uninvaded host cells, suggesting that invaded cells remain in symplastic continuity with surrounding cells and that these surrounding cells are prepared for the subsequent invasion [28]. The invaded cells appear to have lost viability by the time when the IH move into neighbor cells. It has been suggested that host vacuole maintenance is important for successful invasion by *M. oryzae* [18].

Here we describe a live cell imaging method to provide insights into the dynamics of cell death using live-cell confocal microscopy of rice sheath cells mechanically damaged or invaded by fluorescently-tagged *M. oryzae* together with FDA and PI. Using this method, we have demonstrated that initially invaded rice cells are viable but lose viability when the fungus moves into adjacent cells. In addition, this method has revealed unexpected changes of FDA staining patterns in both wound- and pathogen-induced death of rice cells. This allows us to hypothesize the sequence of cytological events leading to plant cell death during the colonization of susceptible rice cells by *M. oryzae*.

## Results and discussion

### Dual staining of rice cells with FDA and PI

To determine FDA staining patterns in rice cells, we used rice sheaths trimmed by hand [12] and FDA working solution (2 μg/ml, 0.2% acetone) as the mounting agent. Confocal microscopy of FDA-stained sheaths revealed bright green fluorescence adjacent to cell walls, associated with nuclei, and as thin strands of fluorescence in epidermal cells and underlying mesophyll cells (Fig. 1b). These patterns are consistent with fluorescein localized in the cytoplasm and nucleoplasm of viable plant cells that contain a large central vacuole, resulting in the thin layer of the cytoplasm adjacent to cell walls and cytoplasmic strands traversing the vacuole [19, 23, 29]. Since nonfluorescent FDA is converted by intracellular esterases into fluorescein analogs exhibiting green fluorescence (Fig. 1a) [20, 21], the bright fluorescent staining in our hand-trimmed sheath strip confirms that the cells in the epidermal layer and one or two layers of mesophyll cells remained viable and metabolically active.

**Fig. 2:**
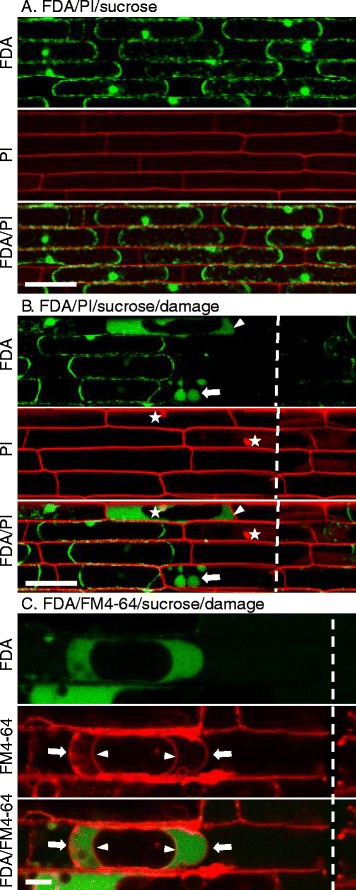
Novel fluorescein patterns in the cytoplasm of cells next to directly damaged cells. **a** Confocal image showing dual staining with FDA (green) and PI (red) followed by treatment with 0.5 M sucrose to induce plasmolysis in live cells. Bar = 50 μm. **b** Confocal image showing dual FDA/PI staining followed by treatment with 0.5 M sucrose to induce plasmolysis in live cells, then mechanically damaged with a razor. White dotted lines indicate where the sheath was damaged with the razor. White stars indicate PI stained nuclei. White arrows indicate membrane bound compartments containing fluorescein. Arrowheads indicate fluorescein evenly distributed in the cytoplasm but excluded from the vacuole. Bar = 50 μm. **c** Confocal image of rice cells sequentially treated with FM4-64 for two hours, FDA for 10 min and 0.5 M sucrose for 10 min, followed by mechanical damage with a razor. White dotted lines indicate where the cell was damaged with the razor. FM4-64 stained the plasma membrane (arrow) and the tonoplast (arrowhead), and fluorescein (green) was retained in the cytoplasm. Bar = 10 μm

As a counter stain to FDA, we also stained excised rice sheaths with PI (10 μg/ml). To ensure there was no overlap in fluorescence detection between FDA and PI, we first performed individual staining with each dye and imaged with detection configured for both. FDA staining alone produced no detectible red fluorescence, while PI staining alone produced no detectible green fluorescence (data not shown). This indicated that green fluorescence was specific to fluorescein, red fluorescence was specific to PI, and autofluorescence was not being collected in either channel. Upon dual FDA/PI staining, cytoplasmic fluorescein was observed in both epidermal and immediately underlying mesophyll cells, and PI fluorescence was observed in cell walls but not in nuclei (n > 300) (Fig. 1b). Lack of PI staining in nuclei indicated that the top two cell layers were viable in our hand-trimmed sheaths, consistent with the FDA straining result. Viability of rice cells has been routinely determined by a sucrose-induced plasmolysis assay, which is marked by the retraction of the plasma membrane from the cell wall in viable cells [12, 13]. To ensure that rice cells displaying cytoplasmic fluorescein and lack of PI staining in nuclei were consistent with the plasmolysis-based assay, we treated rice sheaths with 0.5 M sucrose after dual staining with FDA/PI. All cells, exhibiting both cytoplasmic fluorescein and nuclei absent of PI, plasmolyzed within 10 min of the sucrose treatment (n > 150) (Fig. 2a), consistently confirming the viable rice cells.

### Kinetics of FDA staining

To determine the rate at which fluorescence developed after FDA staining and the duration for which it was detectible, excised sheaths were mounted in FDA working solution and analyzed by time-course confocal microscopy. The intensity of fluorescein within selected cytoplasmic regions was tracked in order to compare fluorescence intensity over time. On average, intensity peaked at 15 min and then slowly declined (Fig. 1c). Although the intensity of fluorescein reduced to approximately one half of peak intensity after 90 min, it was still detectable for many hours after staining by adjusting confocal settings to increase fluorescence detection sensitivity (data not shown). The rapid accumulation and persistence of fluorescein in the rice cytoplasm makes FDA a convenient dye to handle and use for live-cell fluorescence microscopy.

### Novel fluorescein patterns in mechanically wounded rice cells

To determine how fluorescein and PI patterns would change in response to wounding, we used a razor blade to introduce nicks in a FDA/PI-stained sheath. Addition of 0.5 M sucrose caused the protoplast to pull away from the cell wall in fluorescein-stained viable cells (Fig. 2b). Most of the directly damaged cells completely lacked fluorescein, and nuclei of these cells were stained with PI (n > 100), indicating the loss of the viability (Fig. 2b). Occasionally, membrane-bound spherical compartments containing fluorescein were observed in directly damaged cells (*n* = 8) (Fig. 2b). Unexpectedly, we also observed intriguing fluorescein patterns in cells abutting directly damaged cells (*n* = 15) (Fig. 2b). These patterns include: (1) homogeneous distribution of fluorescein throughout the increased area of the cytoplasm with the concomitantly decreased vacuole; (2) the increase of the fluorescein intensity likely due to hydrolysis of more FDA by increased esterase activity or alternatively non-biological hydrolysis of FDA that can occur at low pH [20]; and (3) containment of the brighter fluorescein only in an affected cell likely due to closure of plasmodesmata. We refer to these patterns as novel fluorescein patterns in this study because they differed from typical cytoplasmic fluorescein observed in viable epidermal cells with a large central vacuole (Fig. 1b and 2a) and from patterns described in previous reports [23, 24, 29, 30]. Using FM4-64 that stains both the plasma membrane and the vacuolar membrane [31], we confirmed that fluorescein exhibiting a novel pattern was excluded from the vacuole (n > 200) (Fig. 2c).

Cells that displayed a novel fluorescein pattern often contained a PI-stained nucleus and failed to plasmolyze (*n* = 22) (Fig. 2b). Positive staining of both cytoplasmic fluorescein and nuclear PI seems contradictory because the fluorescein is retained only in the intact membrane, and the appearance of PI-stained nuclei indicates loss of membrane integrity. However, this may be explained by a difference in rates of diffusion for fluorescein and PI across a partially permeabilized plasma membrane. We speculate these cells were in the process of cellular dismantling with the loss of membrane integrity, gradually allowing both PI to enter the cell and fluorescein to diffuse out (Fig. 2b). Taken together, our results show that dual FDA/PI staining is a robust viability assay for rice sheath cells and also that novel fluorescein patterns can be an indicator of cytological events that occur during cell death such as vacuolar shrinkage.

### FDA indicates viability of rice cells infected with rice blast fungus

To evaluate FDA staining during successful fungal infection, we inoculated rice sheaths with a transgenic strain of *M. oryzae* CKF1997. This strain constitutively expresses cytoplasmic red fluorescent protein, allowing simultaneous visualization of fungal hyphae (red) and fluorescein (green) in rice cells when analyzed by confocal microscopy.

**Fig. 3:**
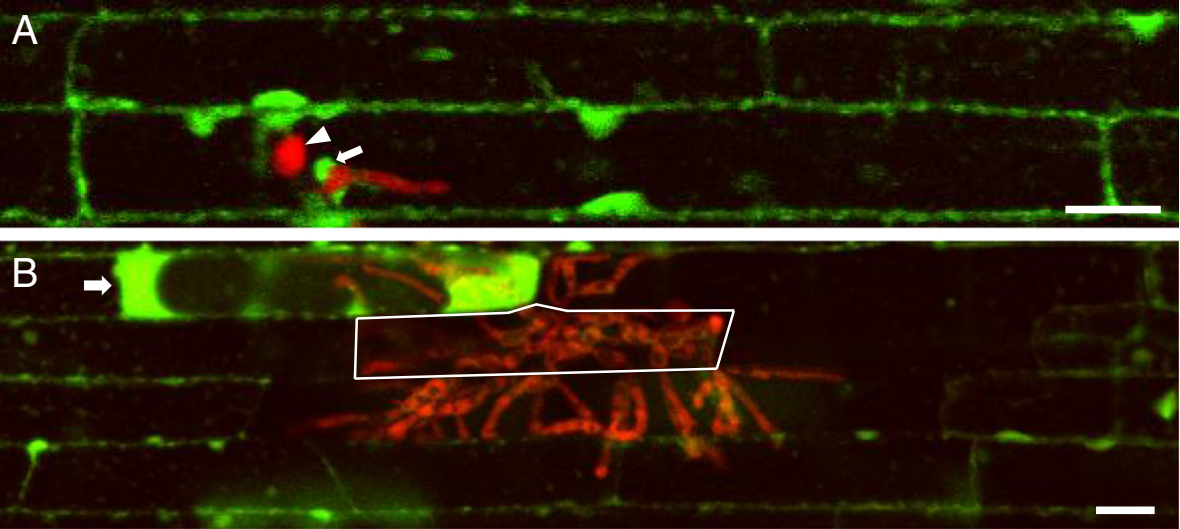
Host cell viability at early and late stages of rice blast invasion. **a** Single plane confocal image of rice sheath epidermal cells infected with *M. oryzae* transformant CKF1997 expressing cytoplasmic tdTomato (shown in red) at 28 hpi and stained with FDA (green). The appressorium (arrowhead) mediated penetration of the host cell and produced IH. Fluorescein is localized in the cytoplasm of both infected and non-infected cells and also associated with a BIC (arrow). **b** Maximum projection of three successive z-stack images covering 6 μm, showing rice sheath epidermal cells infected with *M. oryzae* transformant CKF1997 at 48 hpi and stained with FDA. IH (red) had spread into two cells away from the initially invaded cell indicated with solid white outline. Newly invaded- and non-invaded cells were stained with fluorescence, whereas fully invaded and some partially invaded cells lacked fluorescein. A novel fluorescein pattern (brighter fluorescence in the enlarged cytoplasm) was observed in a partially invaded cell (white arrow). Bars = 20 μm

At an early stage of infection (~28 h post inoculation, hpi), the fungus had penetrated into epidermal cells via an appressorium and subsequently produced IH. Upon staining with FDA, we observed typical cytoplasmic fluorescein in both invaded and uninvaded cells (*n* = 33 infections) (Fig. 3a), confirming previous reports that host cells at this stage were viable, and hyphal invasions were biotrophic [12, 13]. In addition, we observed fluorescein associated with BICs at early stages of host cell invasion (*n* = 30) (Fig. 3), consistent with previous observations that BICs are surrounded by the host cytoplasm [18, 28].

At a later stage of infection (~48 hpi), IH had spread two to three cells away from the initially invaded cell, and we observed the coexistence of both live and dead host cells (*n* = 28 infections) (Fig. 3b). Typical cytoplasmic fluorescein in some invaded cells at the margin of the infection zone or a lack of fluorescein in partially or fully colonized cells was representative of the hemibiotrophic lifestyle of *M. oryzae*, in which live host cells are invaded but then killed by the time IH spread to neighboring cells [13]. Intriguingly, many partially invaded cells displayed novel fluorescein patterns such as brighter fluorescence in the enlarged cytoplasm (*n* = 65) (Fig. 3b) that we observed in mechanically wounded cells (Fig. 2b and c). This suggests that plant cell death resulting from successful invasion by *M. oryzae* and by mechanical damage involves similar morphological features. Further investigation into processes that give rise to novel fluorescein patterns may provide new insight into cytological responses and modes of cell death.

### Time-lapse imaging of rice blast invasion

**Fig. 4:**
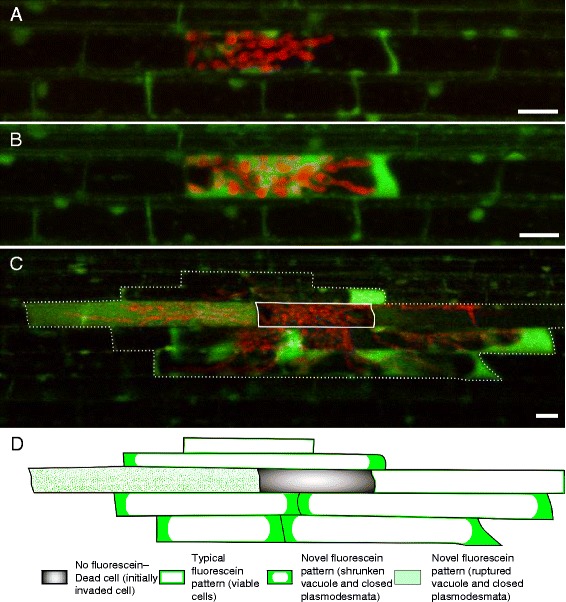
Time-course of the dynamics of host cell death during rice blast invasion. **a** Confocal image showing *M. oryzae* CKF1997 (red) infection in a rice sheath epidermal cell at 30 hpi. Rice cells contained typical cytoplasmic fluorescein. **b** The same infection in (**a**) imaged 2 h later showing a novel fluorescein pattern (brighter fluorescence in the enlarged cytoplasm). **c** The same infection in (**b**) imaged 15 h later. The sample was stained again with FDA to renew fluorescein. The first invaded cell (solid white outline) lacked fluorescein, indicating it was dead by this time. Partially invaded cells showed either typical cytoplasmic fluorescein or novel fluorescein patterns. Dotted white line outlines the cells (total nine cells) infected by hyphae. Bars = 20 μm. **d** Schematic representation of infected cells in (**c**) with fluorescein pattern classification

To determine the dynamics of host cell death during rice blast invasion, we performed time-course confocal microscopy on rice sheaths infected with *M. oryzae* CKF1997 (*n* = 3). We stained infected tissue with FDA once the majority of the initial cell was colonized (30 hpi). Similar to infections at ~28 hpi (Fig. 3a), an initially invaded cell displayed typical cytoplasmic fluorescein patterns (Fig. 4a). After 2 h (32 hpi), the same infection site that was further colonized by IH displayed the novel fluorescein pattern (Fig. 4b). When the same infection site was observed the next day (48 hpi), IH had spread into up to two subsequent neighboring cells. Hyphal growth did not seem to have been affected by FDA when compared to control infection, but fluorescein was rarely detectable (data not shown). Because the fluorescein intensity reduces over time due to its instability in aqueous solution [32], we stained the same sheath again by mounting it in a freshly prepared FDA working solution. This resulted in replenished fluorescein in the infected rice sheath, and we were able to observe both typical and novel fluorescein patterns (Fig. 4c), consistent with previous results of infections at 48 hpi (Fig. 3b). We also observed homogenous fluorescein within an entire invaded cell (Fig. 4c), suggesting the vacuole had ruptured. Time-lapse imaging suggested that the FDA staining pattern in the infected host cell progressed from typical cytoplasmic localization (live cell with the intact vacuole), to novel patterns (dying cell with closed plasmodesmata with the shrunken or ruptured vacuole), to lack of fluorescence (dead cell). Taken together, our results show that fluorescein dynamically stains plant cells during cell death resulting from various stimuli.

## Conclusion

We have developed a fluorescence imaging method to visualize the dynamics of rice cell death in response to mechanical wounding or fungal invasion. This method makes use of the combination of (a) live-cell confocal microscopy, (b) optically transparent rice sheath cells, (c) fluorescently-tagged *M. oryzae*, and (d) fluorescent vital dyes PI and FDA. In particular, we found that FDA is a useful investigational tool for time-course imaging not only for cell viability but also for host vacuolar dynamics during fungal invasion. In addition, this method has revealed unexpected changes of fluorescein patterns during wound- and pathogen-induced death of rice cells. This allows us to hypothesize the sequence of cytological events leading to rice cell death during the colonization of susceptible rice cells by *M. oryzae* IH: (i) live host cell with the intact vacuole, (ii) shrinkage of the vacuole, increase of esterase activity, and closure of plasmodesmata, (iii) collapse of the vacuole, and (iv) death of the infected cell. This method can be used to compare and contrast host cell death associated with disease resistance and susceptibility in rice-*M. oryzae* and other host-pathogen interactions.

## Methods

### Plant and fungal strains

Rice (*Oryza sativa*) strain YT16 was grown under long day conditions (14/10 h, day/night) in a Conviron PGW36 growth chamber with daytime temperature of 28°C and nighttime temperature of 24 °C. Plants were grown in 4” pots with Fanford 3B soil mix. Iron chelate solution (3.25 % iron chelate in water) was added at the time of planting, then 20-10-20 peat lite fertilizer was applied once a week. We generated *M. oryzae* transgenic strain CKF1997 by transforming *M. oryzae* wild-type strain O-137 with the plasmid pCK1292 using Agrobacterium-mediated transformation [33]. pCK1292 was produced by cloning of the tdTomato gene from pAN582 [34] under control of the constitutive promoter from the *M. oryzae* ribosomal protein 27 gene in the binary vector pBGt [35].

### Infection assay

Rice sheath inoculations were performed as previously described [13]. Briefly, excised leaf sheaths (5–9 cm long) from 17- to 21-day old plants were inoculated with a spore suspension (2 x 10^4^ spores/ml in sterile water). The inoculated sheaths were hand-trimmed at 22–28 hpi and immediately used for straining or confocal microscopy.

### Staining procedures and plasmolysis

Fluorescein diacetate (FDA; catalog No. F7378, 5 g power; Sigma) was dissolved in acetone to a stock concentration of 1g/ml. A working solution (2 μg/ml) of FDA was prepared by diluting 2 μl of the stock solution in 1ml of water. A 10 μg/ml working solution of PI (catalog No. P3566; 10 ml of 1 mg/ml solution in water; ThermoFisher) was prepared by diluting 10 μl of the stock solution in 1 ml of water. A dual FDA/PI working solution was prepared by mixing 2 μl of the FDA stock solution and 10 μl of the PI stock solution in a final volume of 1ml water. A 17 mM stock solution of FM4-64 (catalog No. T13320; 10 x 100 μg; ThermoFisher) was prepared by dissolving 100 μg in 9.2 μl water. A working solution of 17 μM was prepared by diluting 1 μl of the stock solution in 1 ml of water. Plasmolysis was performed by submerging sheaths in 1 ml of 0.5 M sucrose for 10 min, then mounting sheaths in the same sucrose solution.

### Confocal microscopy and image analysis

Confocal microscopy was performed with a Zeiss LSM 510 Meta laser scanning confocal microscope. Fluorescein was excited using a 488 nm laser and emission collected between 505 and 530nm, and tdTomato was excited using a 543nm laser and emission detected between 560 and 615 nm. PI was excited using a 543 nm laser and emission collected with the 615 nm long pass filter. Images were processed using the Zen software (Black edition, version 10.0, Zeiss).

### Availability of data and materials

The data sets supporting the results of this article are included within the article.

## Abbreviations

BIC: biotrophic interfacial complex
EIHM: extra-invasive hyphal membrane
FDA: fluorescein diacetate
hpi: hours post inoculation
IH: invasive hyphae
PCD: programmed cell death
PI: propidium iodide
